# Supplementary material for: Identification of Messenger RNA Signatures in Age-Dependent Renal Impairment
Source: Diagnostics (Basel). 2023 Dec 13;13(24):3653. doi: 10.3390/diagnostics13243653 (PMC10742943; doi:10.3390/diagnostics13243653)
Supplement: Supplementary file 1 [file diagnostics-13-03653-s001.zip › Table.pdf]

Table S1. The mRNAs whose expressions were significantly higher (by >2.0 times) in SAMP1-50wk mouse kidneys compared to SAMR1-10wk, SAMR1-50wk, and SAMP1 50wk mouse kidneys by microarray

Gene symbol	GenBank accession no.	Chromosome no.	SAMR1-10wk vs. SAMR1-50wk	SAMR1-10wk vs. SAMP1-10wk	SAMR1-10wk vs. SAMP1-50wk	p-value, ANOVA
A530032D15Rik	NM_213615	chr1	1.27	−1.16	2.99	<0.01
Acox2	NM_053115	chr14	1.08	−1.75	−3.81	<0.01
Apoc2	NM_001277944	chr7	−1.13	−1.00	2.24	0.019
Batf	NM_016767	chr12	1.17	1.05	3.03	<0.01
Card11	NM_175362	chr5	−1.02	1.10	2.48	<0.01
Cd2	ENSMUST00000029456.4	chr3	1.05	1.42	5.34	<0.01
Cd19	NM_001357091	chr7	−1.32	−1.19	5.00	<0.01
Cd37	NM_001290802	chr7	1.39	1.28	2.81	<0.01
Cd52	NM_013706	chr4	1.43	1.65	3.39	<0.01
Cd79b	NM_008339	chr11	1.73	1.23	4.45	<0.01
Cd83	NM_009856	chr13	1.15	1.23	2.70	0.016
Ceacam16	NM_001033419	chr7	−1.28	−2.85	2.18	<0.01
Cenpf	NM_001081363	chr1	−1.94	−1.94	−5.63	<0.01
Comp	NM_016685	chr8	1.93	1.79	7.20	<0.01
Csf2rb2	NM_007781	chr15	1.20	1.13	2.44	<0.01
Csprs	NM_033616	chr8	1.81	1.48	4.44	<0.01
Dock2	ENSMUST00000157036.1	chr11	−1.14	−1.01	2.57	<0.01
Dtx1	NM_008052	chr5	1.07	1.11	2.29	0.017
Fcmr	NM_026976	chr1	2.15	1.53	7.12	<0.01
Gm10050	AK047630	chr15	−1.35	−1.79	−3.61	<0.01
H2-Oa	NM_008206	chr17	1.56	2.03	4.71	<0.01
Ikzf3	ENSMUST00000140876.1	chr11	1.78	1.60	5.39	<0.01
IL-1b	NM_008361	chr2	−1.24	−1.84	3.26	<0.01
LOC664787	NR_131980	chr8	2.99	1.47	10.2	<0.01

Ltb	NM_008518	chr17	1.46	1.56	5.13	<0.01
Lyz1	NM_013590	chr10	1.38	1.48	3.06	<0.01
Mir142hg	AK020764	chr11	1.06	−1.08	2.16	<0.01
Mmp3	NM_010809	chr9	−1.73	−1.28	7.39	<0.01
Ms4a1	NM_007641	chr19	2.42	−1.51	17.2	<0.01
Ncf1	NM_001286037	chr5	−1.12	1.03	2.06	<0.01
Npy6r	NM_010935	chr18	−2.01	−2.43	−14.6	<0.01
Nr4a1	NM_010444	chr15	1.23	1.38	5.49	<0.01
Pcdh7	NM_018764	chr5	−1.34	−2.59	−8.39	<0.01
Pla2g2d	NM_011109	chr4	1.51	−1.00	3.69	<0.01
Runx3	NM_001369050	chr4	1.03	1.11	3.80	<0.01
Samsn1	NM_023380	chr16	1.21	1.38	3.24	0.015
Serpina3n	NM_009252	chr12	1.81	1.73	11.5	<0.01
Slc7a12	NM_080852	chr3	−1.06	−1.94	2.63	<0.01
S100g	NM_009789	chrX	−1.51	−1.64	−5.18	<0.01
Tnfrsf13c	NM_0028075	chr15	1.01	−1.14	18.3	<0.01
Wfdc3	NM_027961	chr2	−1.30	1.12	−3.14	<0.01
Zap70	NM_001289765	chr1	1.03	1.99	4.43	<0.01
4931414P19Rik	NM_028890	chr14	−1.03	−1.18	−4.88	0.013
4921532D01Rik	NM_028890	chr14	−1.27	−1.29	−3.36	<0.01

The data of the SAMR1-10wk group are used as the reference value (1.0). Acox2: acyl-Coenzyme A oxidase 2, branched chain, Apoc2: apolipoprotein C-II, Batf: basic leucine zipper ATF like transcription factor, Card11: caspase recruitment domain family, member 11, Cenpf: centromere protein F, Comp: cartilage oligomeric matrix protein, Csf2rb2: colony stimulating factor 2 receptor, beta 2, Csprs: component of Sp100-rs, Dock2: dedicator of cyto-kinesis 2, Dtx1: deltex 1, Fcmr: Fc mu receptor, Ikzf3: IKAROS family zinc finger 3, IL-1b: interleukin-1b, Ltb: lymphotoxin b, Lyz1: lysozyme 1, Mir142hg: mir142 host gene, mmp3: matrix metallopeptidase, mRNA: messenger RNA, Ms4a1: membrane-spanning 4-domains, subfamily A, member 1, Ncf1: neutrophil cytosolic factor 1, Npy6r: neuropeptide Y receptor Y6, Nr4a1: nuclear receptor subfamily 4, group A, member 1, Pcdh7: Protocadherin 7, Pla2g2d: phospholipase A2 group IID, Runx3: runt related transcription factor 3, S100g: S100 calcium binding protein G, SAMP: senescence-accelerated mouse prone, SAMR: senescence-accelerated mouse resistant, Samsn1: SAM domain, SH3 domain and nuclear localization signals, 1, Serpina3n: serine (or cysteine) peptidase inhibitor, clade A, member 3N, Slc7a12: solute carrier family 7 member 12, Tnfrsf13c: tumor necrosis factor receptor superfamily member 13c, Wfdc3: WAP four-disulfide core domain 3, wk: weeks old, Zap70: zeta-chain associated protein kinase.

Table S2. List of mRNAs differentially expressed in the kidneys of SAM mice and associated known signaling pathways

Pathway	Gene symbol	SAMR1-10wk vs. SAMR1-50wk	SAMR1-10wk vs. SAMP1-10wk	SAMR1-10wk vs. SAMP1-50wk	p-value, ANOVA
Alzheimer's disease	IL-1b	−1.24	−1.84	3.26	<0.01
Burn wound healing	IL-1b	−1.24	−1.84	3.26	<0.01
Cytokines and inflammatory response	IL-1b	−1.24	−1.84	3.26	<0.01
IL-1 signaling pathway	IL-1b	−1.24	−1.84	3.26	<0.01
Immune response in Tg26 glomeruli	IL-1b	−1.24	−1.84	3.26	<0.01
Lung fibrosis	IL-1b	−1.24	−1.84	3.26	<0.01
Mapk signaling pathway	IL-1b	−1.24	−1.84	3.26	<0.01
Myometrial relaxation and contraction pathways	IL-1b	−1.24	−1.84	3.26	<0.01
Toll-like receptor signaling pathway	IL-1b	−1.24	−1.84	3.26	<0.01
Type II interferon signaling	IL-1b	−1.24	−1.84	3.26	<0.01
Tyrobp causal network in microglia	Cd37	1.39	1.28	2.81	<0.01
Nuclear_Receptors	Nr4a1	1.23	1.38	5.49	<0.01
Spinal cord injury	Nr4a1	1.23	1.38	5.49	<0.01
Delta-Notch signaling pathway	Dtx1	1.07	1.11	2.29	0.02
Notch signaling pathway	Dtx1	1.07	1.11	2.29	0.02
Focal adhesion PI3K-Akt-mTOR_signaling_pathway	Comp	1.93	1.79	7.20	<0.01

The data of the SAMR1-10wk group are used as the reference value (1.0). Comp: cartilage oligomeric matrix protein, Dtx1: deltex 1, IL-1b: interleukin-1b, mRNA: messenger RNA, Nr4a1: nuclear receptor subfamily 4, group A, member 1, SAMP: senescence-accelerated mouse prone, SAMR: senescence-accelerated mouse resistant, wk: weeks old.
